# The cytokine storm and thyroid hormone changes in COVID-19

**DOI:** 10.1007/s40618-021-01506-7

**Published:** 2021-02-09

**Authors:** L. Croce, D. Gangemi, G. Ancona, F. Liboà, G. Bendotti, L. Minelli, L. Chiovato

**Affiliations:** 1 Unit of Internal Medicine and Endocrinology, Laboratory for Endocrine Disruptors, Istituti Clinici Scientifici Maugeri IRCCS, 27100 Pavia, Italy; 2 PHD Course in Experimental Medicine, University of Pavia, 27100 Pavia, Italy; 3 Department of Internal Medicine and Therapeutics, University of Pavia, Via S. Maugeri 4, 27100 Pavia, Italy; 4 Postgraduate School in Endocrinology and Metabolism, University of Pavia, 27100 Pavia, Italy

**Keywords:** COVID-19, Cytokine storm, Thyroid, ACE2, Thyroiditis

## Abstract

**Background:**

COVID-19 is now a worldwide pandemic. Among the many extra-pulmonary manifestations of COVID-19, recent evidence suggested a possible occurrence of thyroid dysfunction.

**Purpose:**

The Aim of the present review is to summarize available studies regarding thyroid function alterations in patients with COVID-19 and to overview the possible physio-pathological explanations.

**Conclusions:**

The repercussions of the thyroid of COVID-19 seem to be related, in part, with the occurrence of a “cytokine storm” that would, in turn, induce a “non-thyroidal illness”. Some specific cytokines and chemokines appear to have a direct role on the hypothalamus–pituitary–thyroid axis. On the other hand, some authors have observed an increased incidence of a destructive thyroiditis, either subacute or painless, in patients with COVID-19. The hypothesis of a direct infection of the thyroid by SARS-Cov-2 stems from the observation that its receptor, ACE2, is strongly expressed in thyroid tissue. Lastly, it is highly probable that some pharmaceutical agents largely used for the treatment of COVID-19 can act as confounding factors in the laboratory evaluation of thyroid function parameters.

## Introduction

Ten months after the first report of pneumonias of unknown origin in Wuhan (China) [1], the Coronavirus Disease 2019 (COVID-19), caused by the respiratory syndrome coronavirus 2 (SARS-CoV-2), resulted in a world-spread pandemic. As of November 2020, the number of confirmed cases of COVID-19 has exceeded 35 million worldwide, with more than 1 million COVID-19-related fatalities. The epidemic has put public health systems under severe strain and lead to establishing various degrees of socio-economic lockdowns, both in the developing world and in western countries.

The clinical presentation of COVID-19 patients can vary remarkably, going from completely asymptomatic forms to extremely severe, multisystem clinical involvement. The most common presenting symptoms are due to lung and systemic involvement, and include fever, fatigue and dry cough that can rapidly evolve toward respiratory failure and acute respiratory distress syndrome (ARDS), requiring intensive care support. Less commonly, COVID-19 patients can present a variety of non-pulmonary manifestations, including neurological disorders (both central and peripheral), cardiac abnormalities (including heart failure and arrhythmias), renal failure, liver disease, rhabdomyolysis, coagulopathy and thrombosis [2]. Among the many extra-pulmonary manifestations, researchers have sought the possible occurrence of thyroid dysfunction. Up to now, very few studies have tackled this issue, and there is evidence of discrepancies among different clinical settings. Aim of the present review is to summarize available studies regarding thyroid function alterations in patients with COVID-19 and to overview the possible physio-pathological explanations.

## The cytokine storm induced by SARS-Cov-2 infection

The term “cytokine storm syndrome” describes a clinical syndrome that can occur in patients with severe COVID-19 disease, being characterized by a fulminant and often fatal hyper-cytokinemia leading to multi-organ failure [3, 4]. The term was originally employed to describe the impressive activation of the immune system in the context of graft-versus-host disease [5]. Similar conditions were also described in other pathologic conditions, both infectious (i.e., avian H5N1 influenza virus infection [6] and SARS-Cov-1 infection) and non-infectious (i.e., leukemia patients receiving engineered T cell therapy). The widespread use of the term “cytokine storm” is probably due to its immediate meaning, which actually recalls the role of the immune system in producing an uncontrolled inflammatory response that is detrimental to host cells. Nevertheless, there is still no consensus regarding the exact definition of “cytokine storm”. In the case of COVID-19 disease, the cytokine storm could be the pathogenic process leading to ARDS, which characterizes the most severe cases [7, 8]. ARDS is a devastating event, with an estimated mortality of approximately 40%, defined as lung edema (not explained by cardiac failure or fluid overload) and acute onset of bilateral infiltrates, which result in severe hypoxemia [9, 10]. The exact physio-pathologic mechanisms underlying COVID-19 related cytokine storm are not fully understood; however, data from recent in vitro and in vivo studies and evidence coming from other coronaviruses (such as SARS and MERS) suggest an inflammatory vicious cycle that derives both from the direct cytotoxic effect of the virus on target cells and from the activation of immune cells. [11]. SARS-Cov-2, similarly to SARS-CoV and MERS-CoV viruses, uses the angiotensin-converting enzyme-related carboxypeptidase (ACE-2) receptor to infect target cells [12]. In addition to furin pre-cleavage, the cellular serine protease TMPRSS2 is also required to properly process the SARS- CoV-2 spike protein and facilitate host cell entry. When SARS-CoV-2 infects ACE-2-expressing cells, such as pneumocytes, the active replication and release of the virus can cause abrupt cell damage. This process is called pyroptosis, an abrupt inflammatory form of programmed cell death that leads to the subsequent release of intracellular molecules, including ATP, nucleic acids and damage-associated molecular patterns (PAMPs). These mediators are recognized by nearby endothelial and epithelial cells and alveolar macrophages, triggering the production of pro-inflammatory cytokines, in particular IL-1 β. Using a variety of pattern- recognition receptors (PRRs), alveolar epithelial cells and alveolar macrophages detect the released PAMPs, such as viral RNA, and damage- associated molecular patterns (DAMPs), including ATP, DNA and protein oligomers. A wave of local inflammation ensues, involving increased secretion of the pro-inflammatory cytokines and chemokines (i.e., IL-6, IFNγ, MCP1 and CXCL-10) into the blood of affected patients. The secretion of such cytokines and chemokines attracts immune cells, notably monocytes and T lymphocytes, but not neutrophils, from the blood into the infected site. Pulmonary recruitment of immune cells from the blood and the infiltration of lymphocytes into the airways may explain the lymphopenia and increased neutrophil/lymphocyte ratio seen in around 80% of patients with SARS- CoV-2 infection. The ACE-2 is also present in many immune cells, such as macrophages, dendritic cells and monocytes [13, 14]. The direct SARS-Cov-2 infection of these cell subtypes results in their activation and secretion of inflammatory cytokines, such as interleukin-6 (IL-6) [15]. IL-6 is crucially involved in acute inflammation due to its role in regulating the acute phase response [16]. It is produced by almost all stromal cells and by B lymphocytes, T lymphocytes, macrophages, monocytes, dendritic cells, mast cells and other non-lymphocytic cells, such as fibroblasts, endothelial cells, keratinocytes, glomerular mesangial cells and tumor cells [17]. While in most cases, the infection is followed by an efficient defensive immunological response, in some patients the response is dysfunctional, causing a flood cytokines and chemokines in the serum and resulting in severe lung and even systemic damage. In this scenario, IL-6 exerts potent pro-inflammatory activities through binding to both its membrane receptor (mIL6-R) on immune cells and to a soluble receptor (sIL-6R). The activation of mIL6-R leads to pleiotropic effects on both the innate and acquired immune system. IL-6 binding to sIL-6R also forms a dimeric complex that can bind to the surface of any cell, including lung endothelial cells, resulting in the massive secretion of chemotactic molecules such as vascular endothelial growth (VEGF), monocyte chemoattractant protein–1 (MCP-1), CXCL8 and additional IL-6. This phenomenon attracts more immune cells in the infection site, causing an exponential escalation of the inflammatory process, commonly referred to as “cytokine storm”. Moreover, reduced E-cadherin expression and increased secretion of VEGF increase vascular permeability and leakage, which further contribute to the pathogenesis of ARDS [18]. In spite of the many cytokines, such as IL-1β [19–23], IL-10 [7, 19–21, 24], TNF-α: [1, 19, 22, 23, 25–27] and IFNγ [19, 21, 26, 27], and chemokines, such as CXCL8: [7, 19, 21–23, 25, 28–31], CXCL9: [20, 22, 31, 32], CCL5 [24, 25, 30, 33, 34], CCL2 [1, 19, 22–25, 32, 35], CCL20: [24, 36], CCL3: [1, 19, 22–24, 35, 36] and CCL4 [19, 22, 35, 36] involved in the dysfunctional immunologic response in COVID-19 disease (which are summarized in Table 1), the cytokine IL-6 [20–22, 27–29, 33, 36–45] and the chemokine CXCL10 [1, 20, 22–25, 27, 28, 31, 32, 35, 46, 47] have clearly emerged as recurrent markers of disease severity and poor outcome [38, 41, 42, 48].

**Table 1:** Summary of the cytokines and chemokines involved in COVID-19 pathogenesis

*Cytokines*	
IL-1β	Increased in COVID-19 patients compared with controls [19]. Increased in patients with severe disease when compared with those with mild disease [20], increased in the acute phase of multisystem inflammatory syndrome in children (MIS-C) [21], increased release in broncholaveolar lavage fluid [22], overexpressed mRNA by lung macrophages [22, 23]
IL-6	Increased in patients with severe disease when compared with those with mild disease [29, 37–39, 106]. Elevated in late stages of sever COVID-19 [33]. Correlated with disease severity [28, 41], predictor of mortality [42–44], higher in patients requiring ICU admission [41], correlated with RNAemia [101], increased release in broncholaveolar lavage fluid [22], overexpressed mRNA by lung macrophages [22] and pneumocytes [27], increased in the acute phase of multisystem inflammatory syndrome in children (MIS-C) [21, 36]
IL-10	Increased in patients with severe disease when compared with those with mild disease [7, 20, 24], Increased in COVID-19 patients compared with controls [19], increased in the acute phase of multisystem inflammatory syndrome in children (MIS-C) [21]
TNF-alfa	Increased in COVID-19 patients compared with controls [19]. Increased in patients with severe disease when compared with those with mild disease [1, 25]. Up-regulation of the tumor necrosis factor-driven inflammatory response in PBMCs from COVID-19 patients [26], overexpressed mRNA by lung macrophages [22, 23] and pneumocytes [27]
IFNγ	Increased in COVID-19 patients compared with controls [19], Up-regulation of the IFNγ-driven inflammatory response in PBMCs from COVID-19 patients [26], increased in the acute phase of multisystem inflammatory syndrome in children (MIS-C) [21]. Lack of IFN response by lung macrophages [27]
*Chemokines*	
CXCL10 (IP10)	Increased in COVID-19 patients when compared with controls [32], Increased in patients with severe disease when compared with those with mild disease [1, 20, 24, 25, 46, 47], Correlated with disease severity [28], increased release in broncholaveolar lavage fluid [35], overexpressed mRNA by lung macrophages [22, 23] and pnemocytes [27], predictor of mortality [24], overexpression in nasal swabs of COVID-19 patients [31]
CXCL8 (IL-8)	Correlated with disease severity [7, 25, 28, 29], Increased in COVID-19 patients compared with controls [19], increased release in broncholaveolar lavage fluid [22], overexpressed mRNA by lung macrophages [23], increased in the acute phase of multisystem inflammatory syndrome in children (MIS-C) [21, 30], overexpression in nasal swabs of COVID-19 patients [31]
CXCL9 (MIG)	Increased in COVID-19 patients when compared with controls [32], Increased in patients with severe disease when compared with those with mild disease [20], overexpressed mRNA by lung macrophages [22], overexpression in nasal swabs of COVID-19 patients [31]
CCL5 (RANTES)	Increased in patients with severe disease when compared with those with mild disease [24, 25], predictor clinical outcome [33], increased in children with COVID-19 as compared with adults [30, 34]
CCL2 (MCP-1)	Increased in COVID-19 patients when compared with controls [32], increased in patients with severe disease when compared with those with mild disease [1, 24, 25], increased release in broncholaveolar lavage fluid [35], increased in COVID-19 patients compared with controls [19], overexpressed mRNA by lung macrophages [22, 23]
CCL20 (MIP3 α)	Increased in patients with severe disease when compared with those with mild disease [24], increased in the acute phase of multisystem inflammatory syndrome in children (MIS-C) [36]
CCL3 (MIP1α)	Increased in patients with severe disease when compared with those with mild disease [1, 24] increased release in broncholaveolar lavage fluid [35], increased in COVID-19 patients compared with controls [19], overexpressed mRNA by lung macrophages [22, 23], increased in the acute phase of multisystem inflammatory syndrome in children (MIS-C) [36]
CCL4 (MIP1β)	increased release in broncholaveolar lavage fluid [35], Increased in COVID-19 patients compared with controls [19], overexpressed mRNA by lung macrophages [22], increased in the acute phase of multisystem inflammatory syndrome in children (MIS-C) [36]

## Cytokines as the main mediators of the non-thyroidal illness (NTI) syndrome

Alterations in thyroid function parameters, which are commonly referred to as “non thyroidal illness” (or sick euthyroid syndrome, or low T3 syndrome), can be detected in many severe clinical conditions, both acute (sepsis, trauma, acute myocardial infarction) and chronic (severe malnutrition, liver failure, end-stage renal disease requiring hemodialysis, cancer). The most typical alteration is a decrease in serum T3 level, that can be accompanied, or not, by a slight decrease in TSH level and, as the severity and length of the NTI syndrome increases, also in total T4 [49]. The magnitude of TSH and thyroid hormone changes is proportional to the severity of the underlying NTI and these alterations usually recede after the patient has recovered from the causative condition. The NTI syndrome appears to be an adaptive response to reduced tissue metabolism to preserve energy during systemic illnesses. In this scenario, deiodinases, a group of oxidoreductases that catalyze thyroid hormone activation and/or inactivation, creating a potent mechanism that tightly regulates plasma and intracellular levels of thyroid hormone, play a pivotal role in pathogenesis of the NTI syndrome. The activation of the pro- hormone T4 into the biologically active hormone T3 is catalyzed by type1 (D1) and type 2 (D2) deiodinases via outer-ring deiodination [50]. In contrast, type 3 deiodinase (D3) catalyzes the inactivation of both T4 and T3, by promoting the conversion of T4 to reverse T3 and the conversion of T3 to 3,3-T2, both biologically inactive. Thus, D3 contributes to thyroid hormone homeostasis protecting tissues from excess of thyroid hormones. D1 and D2 differ by their kinetic properties, substrate, specificity, and susceptibility to inhibitory drugs, as well as by their response to changes in the thyroid hormone status. While D2 is an exclusive outer-ring deiodinase, D1 promotes inner ring as well as outer-ring deiodination. The highest levels of D1 activity in humans are found in thyroid, liver, and kidney; while D2 is more widely expressed, being found in the pituitary, brain, thyroid, skin, skeletal, and heart muscles [51]. Reduced conversion of T4 to T3, and increased activity of D3 are typically observed in the NTI syndrome [52].

The NTI syndrome was consistently reported in patients admitted to intensive care units (ICU) [53, 54] and in patients with pneumonia [55]. Thus, it appears highly probable that patients experiencing severe COVID-19 disease requiring ICU admission could manifest this syndrome. Although the mechanisms underlying the NTI syndrome are multifactorial, circulating cytokines are considered as its main mediators, due to their multiple effects on the hypothalamic-pituitary thyroid axis, on circulating thyroid hormone binding proteins and on the peripheral metabolism of thyroid hormones [56]. In vitro and in vivo data demonstrating these effects are summarized in Table 2 for four main cytokines: IL-1β [57–69], TNF-α [7, 57, 60, 70–77], IL-6 [74, 78–88] and IFN-γ [77, 89–99].

**Table 2:** Summary of the in vitro and in vivo effects of four main pro-inflammatory cytokines

	Evidence from in vitro data	Evidence from animal models	other interfering elements	Patients data
IL-1 beta	↓ iodide uptake in FRTL-5 and in porcine thyroid cells [57–59] ↓ T3 secretion in human thyrocytes [60], ↓ Tg mRNA expression in human thyrocytes [61, 97] ↓ TPO mRNA in Graves’ thyrocytes [63] ↓ induction of DIO1 mRNA [64] ↓ DIO1 activity in hepatocytes [65, 66] ↓TSH secretion in rat pituitary cells [69]	↓ TT4, TT3, TSH production in rats [67, 68]		
TNF-α	↓ iodide uptake in FRTL-5 and Thyroid cancer cells [Spizweg et al. 1999, 77], ↓ Tg synthesis in human thyrocites [70] ↓ T3 secretion in human thyrocites [60], ↓ DIO1 activity [7, 71], ↓ basal and TSH-stimulated TPO and Tg gene expression in FRTL-5 cells and human normal thyroid cells [77] ↓ TSH synthesis in rat pituitary cells [74]	↓ TSH, TT3, TT4 ↓ hypothalamic TRH syntesis, ↓TSH glycosylation in mouse and rat models [57, 72]	↓ albumin production by rat hepatocytes(Perlmutter et al*.* 1986)	↓ serum T3 in cancer patients treated with recombinant human TNF-α (Feelders et al*.* 1999)
IL-6	↓ iodide uptake in FRTL-5 and thyroid cancer cells ↓ Tg synthesis in human thyrocites ↓ T3 secretion in human thyrocites [78, 79], ↑proliferation in human thyrocites, ↓ DIO1 mRNA [81] ↓ DIO1 and DIO2 activity [81] ↑ DIO3 activity [81] ↓ basal and TSH-stimulated TPO and Tg gene expression in FRTL-5 cells and human normal thyroid cells [80] ↑ expression of DIO2 in rat pituitary cells [74]		↓ hepatic synthesis of TBG, TTR, albumin [82, Ramadori et al*.* 1988)	Inverse correlation between IL-6 and FT3 levels [Bartalena et al. 1994] [Boelen et al. 1993,1995; Davies et al. 1996; Friberg et al. 2002) Intravenous injection of IL-6 given to healthy humans causes a transient decrease in serum T3 and an increase in rT3 [88]
IFN-γ	↑ iodide uptake, ↓thyroglobulin synthesis, ↓basal and TSH-stimulated TPO and Tg gene expression in FRTL-5 cells and human normal thyroid cells ↓proliferation of human thyroid cells ↑ iodide uptake [89], ↓ Tg synthesis [90] ↓ basal and TSH-stimulated TPO and Tg gene expression in FRTL-5 cells and human normal thyroid cells [90–94] ↓ proliferation of human thyroid cells [95] ↓DIO-1 activity in FRTL-5 [77]	Severe hypothyroidism in transgenic mice expressing IFN-γ in thyroid cells due to downregulation inhibition of the NIS gene [96]		No acute effect on circulating TSH, FT3, FT4 levels in critically ill patients after treatment with IFN-γ [98], higher risk of hypothyroidism in patients chronically treated [99]

Although CXCL10 and other IFN-inducible chemokines have been thoroughly studied for their pivotal role in the pathogenesis and maintenance of autoimmune thyroid diseases [100], their role in thyroid function perturbation occurring in critically ill patients is probably negligible. As their name suggests, chemokines act as potent chemo-attractants towards cells that express their surface receptors, mainly belonging to the immune subset. For this reason, chemokines usually exert their action by attracting target cells via a chemical gradient into a specific site. This action is radically different from that of cytokines, which usually have pleiotropic and systemic effects on several cell types. Indeed, no in vitro study has ever highlighted alterations in thyroid hormone production or deiodinase activity after treatment with CXCL10.

## Thyroid function alterations in patients with COVID-19 disease

Six main studies investigated thyroid function in hospitalized patients with Covid 19 disease. Several case reports were also published, mainly in outpatients suffering with subacute thyroiditis.

Chen et al. investigated thyroid function parameters in a group of 50 patients with unremarkable history of thyroid disease hospitalized for COVID-19 (15 mild, 23 severe and 12 critical cases). Two control groups were also investigated: 54 healthy subjects and 50 patients with non-COVID-19 pneumonia of similar severity. A low TSH was present in 56% of COVID-19 patients. COVID-19 patients were also found to have significantly lower serum TSH and total T3 levels as compared both with healthy subjects and with patients affected by non-COVID-19 pneumonia. Moreover, there was a significant association between a trend towards a reduction in serum TSH and total T3 levels and the disease severity. Serum total T4 levels were similar in the three groups. Although these findings were consistent with the development of an NTI syndrome in patients with severe COVID-19 disease, the fact that significant differences occurred between COVID-19 patients and patients with severe pneumonia suggested a specific role of SARS-Cov-2 infection in thyroid function alteration [101].

A completely different thyroid function picture was described by Lania et al. in Italy. These Authors investigated 287 consecutive COVID-19 patients (193 males and 183 females with a median age of 66 years) being hospitalized in a non-intensive care unit. No control group was enrolled. They found that 58 (20.2%) of these patients had serum TSH levels below the reference range, with 31 of them having laboratory evidence of overt thyrotoxicosis and 27 having normal serum FT3 and FT4 levels. Fifteen patients (5.2%) had laboratory data indicating hypothyroidism, which was overt in 3 and subclinical in 13 of them. None of the patients complained of pain in the neck, while a new-onset atrial fibrillation was observed in 10 patients with overt thyrotoxicosis. Five thyrotoxic patients experienced a thromboembolic event (venous thromboembolism in 3 cases, ischemic stroke in 2 cases). No patient tested positive for thyroid autoantibodies. Eight thyrotoxic patients underwent thyroid ultrasound, showing signs of thyroid inflammation in 2 patients, small thyroid nodules in 3 patients and no significant alteration in the remaining 3 patients. In none of the them the classic ultrasound findings of subacute thyroiditis were described. In multivariate analysis, a significant inverse correlation between serum IL-6 and TSH levels was observed. In 7 thyrotoxic patients, thyroid function was longitudinally investigated for a short follow up (median 10 days). A progressive decrease in serum FT4 levels was detected, which was not influenced by methimazole treatment in 2 of them. Based on these findings the Authors hypothesized that their COVID-19 patients experienced a destructive, “silent” thyroiditis [102].

The hypothesis that the thyroid gland could be involved in COVID-19 disease stems from experiences in previous coronavirus pandemics (such as SARS and MERS) and from the potential susceptibility of thyroid cells to SARS-Cov-2 infection. Alterations of both thyroid function and structure were reported in patients affected by SARS-CoV-1. In autopsy specimens of 5 patients died of SARS, an extensive apoptotic process in follicular epithelium, causing exfoliation of epithelial cells into the follicle and alterations in follicular morphology were observed. No inflammatory infiltration was found in any specimen [103]. The same authors published a report regarding the immunohistochemical evaluation of pituitary histology on the same 5 patients, showing that the number and the staining intensity of TSH-expressing cells was remarkably reduced when compared with controls [104]. The anatomic location of the thyroid, which is contiguous to the upper airways, a main entrance site of corona viruses, further supports the hypothesis that the thyroid could be a direct target of SARS-CoV-2. As previously discussed, SARS-COV-2, similarly to the virus that caused SARS, uses the ACE-2 as its cellular entry receptor [105]. In this regard it is important to recall that a recent study demonstrated that ACE-2 in strongly expressed in follicular thyroid cells making them a potential target for SARS-COV-2 entry [106, 107]. In line with this in vitro data, several recent case reports described patients with SARS-Cov-2 infection being diagnosed with typical painful subacute thyroiditis [108–113]. It should be highlighted that these patients suffered with a mild (in 4 cases) or moderate (in 4 cases, requiring hospitalization) COVID-19 disease, but none of them experienced a cytokine storm or required ICU admission. Most of them were female patients (7 out of 8) and their sign and symptoms of subacute thyroiditis occurred between 5 and 36 days (median 19) after the onset of COVID-19 disease. In all cases a TSH value below 0.1 µU/ml at the onset of subacute thyroiditis was observed, while thyroid autoantibodies were undetectable in all cases.

A further study in hospitalized patients provides evidence for the occurrence of a destructive thyroiditis in patients with COVID-19. Muller et al. investigated thyroid function in 85 patients who were admitted to a high intensity care unit (HICU) in 2020 because of COVID-19. Non COVID-19 patients admitted to the same HICU in 2019 and COVID-19 patients admitted to a low-intensity care unit (LICU) in 2020 served as controls. They found that 13 (15%) of 85 patients admitted to the HICU for COVID-19 disease had thyrotoxicosis (defined as TSH < 0.28 mIU/L and/or FT4 > 21.9 pmol/L). As compared with this figure, one (1%) out of 78 non COVID-19 patients hospitalized in 2019 in the same HICU and one (2%) of 41 COVID-19 patients admitted to the LICU were thyrotoxic. Three patients (3.5%) in the COVID-19 group, as compared with 7 (9%) and 4 (9.8%) patients in the non-Covid HICU group and in the LICU group, respectively, were hypothyroid (defined as TSH > 4.30 mIU/L and/or FT4 < 10.3 pmol/L). COVID-19 patients hospitalized in the HICU had lower serum TSH and higher serum FT4 levels than patients in both control groups, while FT3 levels were similarly low in the three groups. In 8 thyrotoxic patients (1 patient with subclinical hypothyroidism, 1 patient with overt hypothyroidism, and 6 thyrotoxic patients) with COVID-19 disease a post-discharge follow-up was available: the 2 hypothyroid patients were still hypothyroid at the initial follow-up. One patient had positive AbTg and AbTPO, while the other had negative thyroid autoantibodies. Both patients had a marked diffuse hypoechoic pattern of the thyroid at ultrasound. The 6 patients with low or suppressed TSH concentrations or thyrotoxicosis at baseline had normal thyroid function and were negative for thyroid autoantibodies at follow-up; none reported neck pain ever. Thyroid ultrasound was performed in 5 of these patients: all of them had a diffuse mild hypoechoic pattern at thyroid ultrasound, while in 3 patients focal markedly hypoechoic areas, typical of subacute thyroiditis, were observed. Such areas corresponded to focal reduced Technetium-99 m uptake at single-photon emission Computed Tomography imaging, and the thyroid gland showed a general low to normal or reduced Technetium- 99 m uptake. The authors described their finding as a combination of thyrotoxicosis (possibly due to a subacute thyroiditis) and NTI syndrome [113]. From a clinical point of view, the fact that some of the classic symptoms of subacute thyroiditis (such as asthenia, fever and neck pain) are shared by COVID-19 patients could suggest that, unless specifically searched for, the thyroid disease might be overlooked. Moreover, the frequent use of corticosteroids such, as dexamethasone, in the therapy of patients with severe COVID-19 could abolish neck pain in those with concomitant subacute thyroiditis.

Compared with the previous studies of Lania et al. [102] and Muller et al. [113], Khoo et al. [114] recently reported different results. The authors described a cohort of 456 hospitalized patients from 3 hospitals in London with a clinical suspicion of COVID-19 in which both TSH and FT4 levels were routinely evaluated. In particular, the authors compared thyroid function parameters between the 334 patients with a confirmed diagnosis of COVID-19 and 122 patients without a COVID-19 diagnosis. Results showed that the vast majority (86.5%) of patients with COVID-19 were euthyroid, while only a minority were subclinical hypothyroid (5.1%) or overt hypothyroid (0.6%). Eight patients had a suspect of secondary hypothyroidism (2.4%). No patient received a diagnosis of neither subclinical nor overt thyrotoxicosis. The distribution of thyroid function alterations was similar between COVID-19 and non-COVID-19 patients. The authors observed slightly lower TSH and FT4 levels among COVID-19 patients when compared with the non-COVID-19 ones, even within the normal range. Moreover, lower TSH and FT4 levels were observed in patients with a fatal disease and in those admitted to ICU. A significant inverse relationship between C-reactive protein and cortisol levels and TSH levels was observed in COVID-19 patients. In a subset of patients where previous evaluations of TSH and FT4 levels were available, a slight reduction in both TSH and FT4 levels was observed in COVID-19 patients, but not in the non-COVID-19 ones. Lastly, among 55 patients in which an evaluation of thyroid function parameters before admission, at the moment of admission and after a median follow-up time of 79 days, was performed, results showed that thyroid function parameters returned to baseline levels after recovery of COVID-19. The authors concluded that in their cohort there was no suggestion of a COVID-19-related thyroiditis/thyrotoxicosis, but that their findings are more indicative of a NTI syndrome. Most importantly, all patients taking corticosteroids either at baseline or during COVID-19 were excluded from this study. One of the limitations of this study is the lack of measurements of thyroid autoantibodies and of FT3 or rT3. On the other hand, the study has the advantage of including patients in whom both TSH and FT4, irrespectively of TSH levels, were evaluated.

In another recent study from Hong Kong, Lui et al. [115] evaluated 191 COVID-19 patients admitted to a non-intensive care unit. Among enrolled patients, 11 cases showed reduced serum TSH levels with normal fT4 and fT3, but in none of them overt thyrotoxicosis was found. Three of these patients also had detectable levels of TSH receptor antibodies (TRAb), suggesting a diagnosis of Graves’ disease. The authors highlighted that a higher SARS-CoV-2 load characterized patients with a reduced TSH. Moreover, an isolated low serum fT3 level was detected in12 other patients, who had higher acute-phase indexes (C-reactive protein levels, erythrocyte sedimentation rate and LDH) levels as compared with the rest of the cohort. Patients with low serum FT3 levels had a higher chance of clinical deterioration during the follow-up. The authors concluded that in their cohort two distinct groups of patients with COVID-19 related thyroid dysfunction could be identified: one characterized by subclinical thyrotoxicosis (mostly related with a thyroiditis process) and one characterized by a low T3 levels (probably due to a NTI syndrome).

In further study by Gao et al. [116], thyroid function parameters were evaluated in a cohort of 100 COVID-19 patients from Wuhan, and findings were compared between critical and non-critical patients. Results showed that TSH and FT3 levels, but not FT4 levels, were significantly lower in critically ill patients when compared with the non-critically ill ones. Moreover, FT3 levels at baseline, but not TSH or FT4, were independent predictors of mortality in this cohort of patients. An inverse correlation between C-reactive protein, TNF-alfa and IL-6 levels and TSH and FT3 levels was observed, while no correlation with FT4 was found. These data strongly suggest the occurrence of a NTI syndrome in this cohort of patients.

Lastly, some anecdotal case report described cases of severe hypothyroidism or Graves’ thyrotoxicosis onset after COVID-19 [117, 118], but no systematic study has evaluated this issue so far.

## Confounding factors: COVID-19 therapies

Among the increasing number of drugs that are or have been recommended for the treatment of COVID-19 patients, some do interfere with the hypothalamic-pituitary thyroid axis or with laboratory tests for the measurement of free thyroid hormones.

### Glucocorticoids

The use of glucocorticoids in COVID-19 patients has been widely debated [119, 120]. In the early phases of the pandemic, many national guidelines either contraindicated or did not recommend glucocorticoid treatment [121]. However, in the clinical practice, almost 50% of COVID-19 patients have been treated with some form of glucocorticoid [122, 123]. Afterwards, a randomized clinical trial provided evidence that treatment with dexamethasone could reduce the 28-day mortality in COVID-19 patients receiving respiratory support, with no benefit (and possible harm) in those who do not require oxygen [124].

**Fig. 1:**
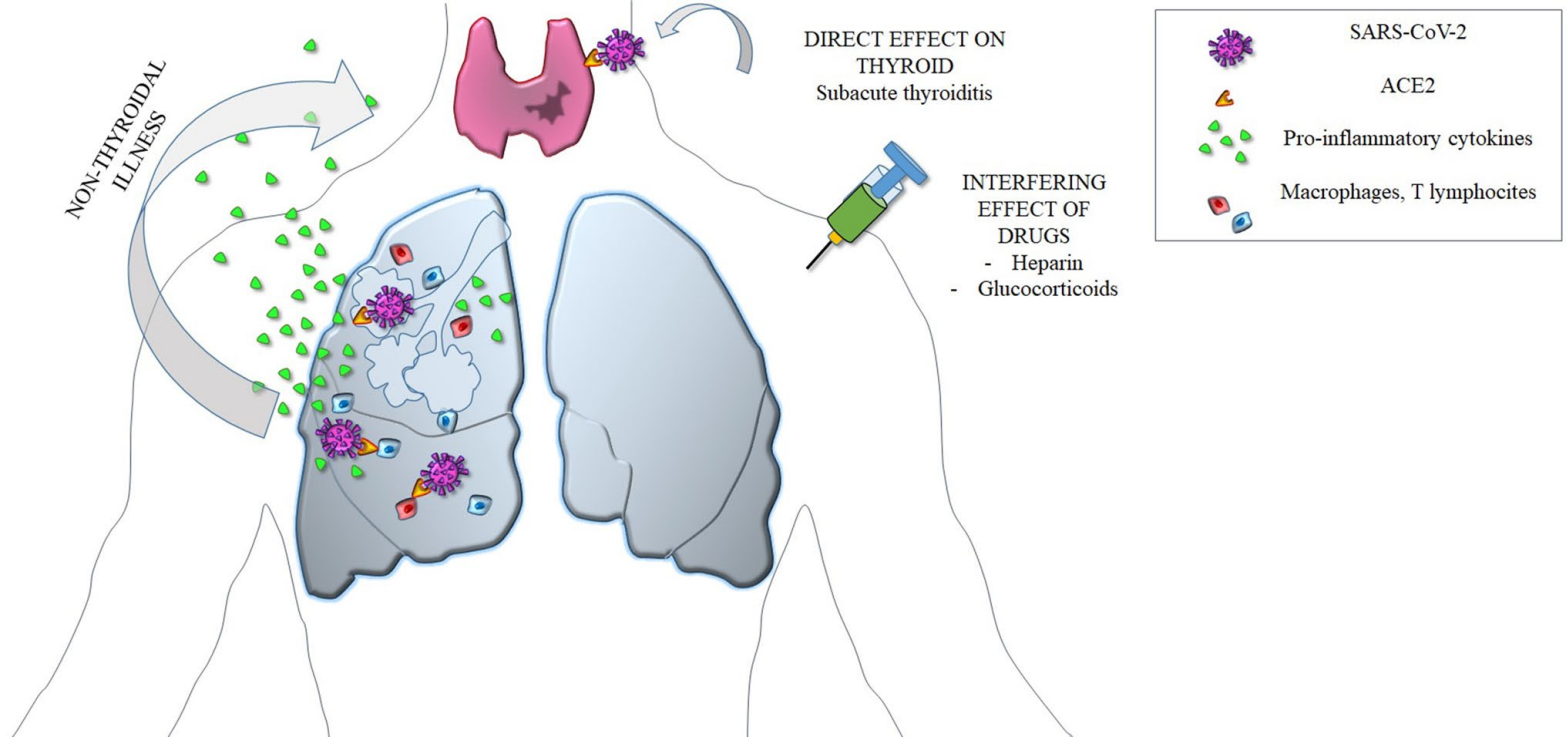
Schematic representation of the possible mechanisms causing alterations in thyroid function parameters in patients with COVID-19

Glucocorticoids have long been known to affect serum TSH levels in humans [125, 126]. Even low doses of dexamethasone can lower serum TSH levels, while higher doses of prednisone are required to reach the same effect [126]. Glucocorticoids appear to suppress release of TSH through a direct inhibitory effect on pituitary thyrotrope cells [127] and an inhibition of TRH release in the hypothalamus [128, 129]. Moreover, glucocorticoids can interfere with the production of active T3, through a direct induction of type 3 deiodinase and an increased conversion of T3 to reverse T3 [130]. Acute administration of glucocorticoids to humans or rats decreases the ratio of circulating T3 to T4, implying that these agents block T4 to T3 conversion. Recent studies in humans indicate that D3 activity is induced by dexamethasone, and the acute decrease in serum T_3_ that follows a high dose of glucocorticoids may be due to an increase in D3-mediated T3 clearance via 5 deiodination [131]. The resulting reduction in T3 levels can mimic a NTI syndrome [132, 133] (Fig. 1).

### Heparin

**Fig. 2:**
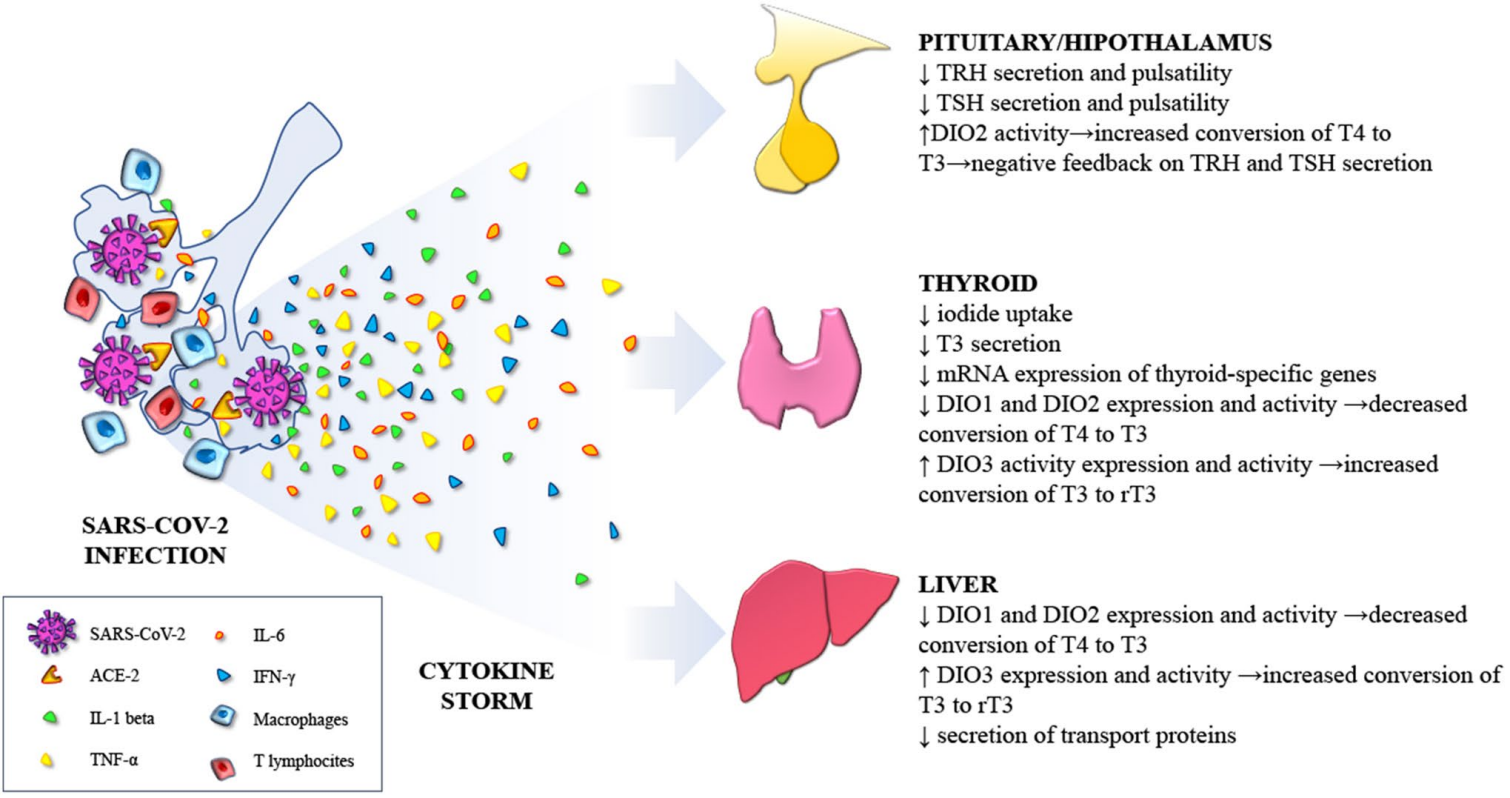
Schematic representation of the mechanisms through which SARS-Cov-2 related cytokine storm can cause a Non-Thyroidal-Illness syndrome in COVID-19 patients

Heparin or low molecular weight heparin (LMWH) is increasingly prescribed in COVID-19 patients. An anti-thrombotic prophylaxis is mandatory in hospitalized and bedridden patients, who are also exposed to an increased pro-thrombotic risk directly related to COVID-19 disease. Moreover, heparin has potential beneficial non-anticoagulant effects, including reduction of endothelial leakage, neutralization of cytokines and chemokines, interference with leukocyte trafficking and with viral cellular entry [134] (Fig. 2).

Unfortunately, heparin is known to interfere in free thyroid hormone assays. Heparin liberates lipoprotein lipase from the vascular endothelium. As consequence, blood samples from heparin-treated patients have increased lipoprotein lipase activity, which persists in vitro and generates non-esterified fatty acids (NEFA) during sample storage or incubation. Free thyroid hormone assays, especially those with prolonged incubation periods, such as measurement by means of equilibrium dialysis, are most affected, since NEFA displace T4 and T3 from binding proteins, causing spuriously high values [135]. The effect is greater if samples are stored for a long time before the assay. Similar effects are seen with LMWH preparations [136]. Standard competitive free hormone assays are generally less affected by this phenomenon, since the incubation period is shorter and occurs at a temperature lower than 37 °C, but the interference cannot be completely excluded neither in this case. If the sample is stored for a long time the amount of NEFA in the samples constitutes an insuperable pre-clinical problem, that can be overcome only adding a non-toxic additive that can block the heparin-induced lipase at the moment of sample collection. If these laboratory alterations are suspected, the assay should be repeated at least 10 h after heparin withdrawal [136]. Moreover, total T4 and total T4 are likely to be more informative in this context [137].

### Combined confounding factors

A study by Sapin et al. demonstrated how multiple inaccuracies of the hormonal thyroid profile can occur in critically ill patients submitted to multiple therapies. These authors evaluated serum FT4 results obtained with 6 different commercial kits in 20 patients who had undergone bone marrow transplantations and who were previously euthyroid. Patients were treated with heparin and glucocorticoids, similarly to what happens in COVID-19 patients. Assay methods that involved sample incubation at 37 °C (such as equilibrium dialysis) gave falsely high FT4 values in 20–40% of patients, while analogue tracer methods, influenced by tracer binding to albumin, gave subnormal estimates of FT4 in 20–30% of them, even if the values were closer to the reference range. By contrast, total T4 was normal in the majority of these presumably euthyroid subjects. Interestingly, marked alterations in serum TSH were found, since half of the subjects had a suppressed serum TSH value. This change was probably attributable to glucocorticoid treatment. It is evident that in this context dosing artefacts in TSH and FT4 could be falsely interpreted as a case of thyrotoxicosis [138].

## Conclusions

Patients with severe COVID-19 disease may undergo the so-called cytokine storm. In vitro studies, experiments in animal models, and evidence in humans indicate that cytokines play an important role in the development of the NTI syndrome observed in critically ill patients. Several studies in hospitalized patients with COVID-19 disease indicate that the NTI syndrome is the most consistently observed alteration of thyroid function parameters. SARS-CoV-2 may also infect the thyroid producing a typical (painful) or, possibly, an atypical (painless) subacute thyroiditis. At present there is no evidence for a direct thyroid cytotoxic effect of cytokines on thyroid cells, at least in humans. Glucocorticoids and heparin, frequently administered to COVID-19 patients, may act as confounding factors due to their effect on the HPT axis (glucocorticoids) and to their interference (heparin) in the assays for free thyroid hormones.

## References

[1] Huang C, Wang Y, Li X (2020). Clinical features of patients infected with 2019 novel coronavirus in Wuhan, China. Lancet.

[2] Mao L, Jin H, Wang M (2020). Neurologic manifestations of hospitalized patients with coronavirus disease 2019 in Wuhan, China. JAMA Neurol.

[3] Tisoncik JR, Korth MJ, Simmons CP, Farrar J, Martin TR, Katze MG (2012). Into the eye of the cytokine storm. Microbiol Mol Biol Rev.

[4] Coperchini F, Chiovato L, Croce L, Magri F, Rotondi M (2020). The cytokine storm in Covid-19: an overview of the involvement of the chemokine/chemokine-receptor system. Cytokine Growth Factor Rev.

[5] Jl F, Abhyankar S, Dg G (1993). Cytokine storm of graft-versus-host disease: a critical effector role for interleukin-1. Transplant Proc.

[6] Yuen KY, Wong SS (2005). Human infection by avian influenza A H5n1. Hong Kong Med J.

[7] Guan WJ, Ni ZY, Hu Y (2020). Clinical characteristics of coronavirus disease 2019 in China. N Engl J Med.

[8] Xu Z, Shi L, Wang Y (2020). Pathological findings of Covid-19 associated with acute respiratory distress syndrome. Lancet Respir Med.

[9] Berlin DA, Gulick RM, Martinez FJ (2020). Severe Covid-19. N Engl J Med.

[10] Bhatia M, Rl Z, Jeyaseelan S (2012). Role of chemokines in the pathogenesis of acute lung injury. Am J Respir Cell Mol Biol.

[11] Moore JB, June CH (2020). Cytokine release syndrome in severe Covid-19. Science.

[12] Turner AJ, Tipnis SR, Jl G, Rice G, Hooper NM (2002). Aceh/Ace2 is a novel mammalian metallocarboxypeptidase and a homologue of angiotensin-converting enzyme insensitive to ace inhibitors. Can J Physiol Pharmacol.

[13] Merad M, Martin JC (2020). Pathological inflammation in patients with Covid-19: a key role for monocytes and macrophages. Nat Rev Immunol.

[14] Webb BJ, Peltan ID, Jensen P (2020). Clinical criteria for Covid-19-associated hyperinflammatory syndrome: a cohort study. Lancet Rheumatol.

[15] Kang S, Tanaka T, Narazaki M, Kishimoto T (2019). Targeting Interleukin-6 signaling in clinic. Immunity.

[16] Brocker C, Thompson D, Matsumoto A, Nebert DW, Vasiliou V (2010). Evolutionary divergence and functions of the human interleukin (Il) gene family. Hum Genom.

[17] Scheller J, Rose-John S (2006). Interleukin-6 and its receptor: from bench to bedside. Med Microbiol Immunol.

[18] Tanaka T, Narazaki M, Kishimoto T (2016). Immunotherapeutic implications of Il-6 blockade for cytokine storm. Immunotherapy.

[19] De Biasi S, Meschiari M, Gibellini L (2020). Marked T cell activation, senescence, exhaustion and skewing towards Th17 in patients with Covid-19 pneumonia. Nat Commun.

[20] Lucas C, Wong P, Klein J (2020). Longitudinal analyses reveal immunological misfiring in severe Covid-19. Nature.

[21] Carter MJ, Fish M, Jennings A (2020). Peripheral immunophenotypes in children with multisystem inflammatory syndrome associated with Sars-Cov-2 infection. Nat Med.

[22] Liao M, Liu Y, Yuan J (2020). Single-cell landscape of bronchoalveolar immune cells in patients with Covid-19. Nat Med.

[23] Rl C, Lukassen S, Trump S (2020). Covid-19 severity correlates with airway epithelium-immune cell interactions identified by single-cell analysis. Nat Biotechnol.

[24] Hue S, Beldi-Ferchiou A, Bendib I (2020). Uncontrolled innate and impaired adaptive immune responses in patients with Covid-19 Ards. Am J Respir Crit Care Med.

[25] Alabi OA, Bakare AA, Xu X, Li B, Zhang Y, Huo X (2012). Comparative evaluation of environmental contamination and dna damage induced by electronic-waste in Nigeria and China. Sci Total Environ.

[26] Lee JS, Park S, Jeong HW (2020). Immunophenotyping of Covid-19 and influenza highlights the role of type I interferons in development of severe Covid-19. Sci Immunol.

[27] Vanderheiden A, Ralfs P, Chirkova T et al (2020) Type I and type III interferons restrict Sars-Cov-2 infection of human airway epithelial cultures. J Virol. 10.1128/Jvi.00985-20 10.1128/JVI.00985-20 PMC7495371 32699094

[28] Rydyznski Moderbacher C, Ramirez SI, Dan JM (2020). Antigen-specific adaptive immunity to Sars-Cov-2 in acute Covid-19 and associations with age and disease severity. Cell.

[29] Agrawal A, Zhuo H, Brady S (2012). Pathogenetic and predictive value of biomarkers in patients with ali and lower severity of illness: results from two clinical trials. Am J Physiol Lung Cell Mol Physiol.

[30] Diorio C, Henrickson SE, Vella LA (2020). Multisystem inflammatory syndrome in children and Covid-19 are distinct presentations of Sars-Cov-2. J Clin Investig.

[31] Lieberman NAP, Peddu V, Xie H (2020). In vivo antiviral host transcriptional response to Sars-Cov-2 by viral load, sex, and age. Plos Biol.

[32] Blanco-Melo D, Nilsson-Payant B, Liu W et al (2020) Imbalanced host response to Sars-Cov-2 drives development of Covid-19. Cell 181(5):1036–1045.E1039. 10.1016/J.Cell.2020.04.026 10.1016/j.cell.2020.04.026 PMC7227586 32416070

[33] Zhao Y, Qin L, Zhang P et al (2020) Longitudinal Covid-19 profiling associates Il-1ra And Il-10 with disease severity and rantes with Mild disease. Jci Insight. 10.1172/Jci.Insight.139834 10.1172/jci.insight.139834 PMC7406242 32501293

[34] Moratto D, Giacomelli M, Chiarini M (2020). Immune response in children with Covid-19 Is characterized by lower levels of T-cell activation than infected adults. Eur J Immunol.

[35] Xiong Y, Liu Y, Cao L (2020). Transcriptomic characteristics of bronchoalveolar lavage fluid and peripheral blood mononuclear cells in Covid-19 patients. Emerg Microbes Infect.

[36] Gruber CN, Patel RS, Trachtman R (2020). Mapping systemic inflammation and antibody responses in multisystem inflammatory syndrome in children (Mis-C). Cell.

[37] Laing AG, Lorenc A, Del Molino Barrio I (2020). A dynamic Covid-19 immune signature includes associations with poor prognosis. Nat Med.

[38] Borges Do Nascimento IJ, Von Groote TC, O'mathúna DP et al (2020) Clinical, laboratory and radiological characteristics and outcomes of novel coronavirus (Sars-Cov-2) infection in humans: a systematic review and series of meta-analyses. Plos One 15(9):E023923510.1371/Journal.Pone.0239235 10.1371/journal.pone.0239235 PMC7498028 32941548

[39] Zeng Z, Yu H, Chen H (2020). Longitudinal changes of inflammatory parameters and their correlation with disease severity and outcomes in patients with Covid-19 from Wuhan, China. Crit Care.

[40] Li S, Jiang L, Li X (2020). Clinical and pathological investigation of patients with severe Covid-19. JCI Insight.

[41] Coomes EA, Haghbayan H (2020). Interleukin-6 in Covid-19: a systematic review and meta-analysis. Rev Med Virol.

[42] Ruan Q, Yang K, Wang W, Jiang L, Song J (2020). Correction to: clinical predictors of mortality due to Covid-19 based on an analysis of data of 150 patients from Wuhan, China. Intensive Care Med.

[43] Zhou F, Yu T, Du R (2020). Clinical course and risk factors for mortality of adult inpatients with Covid-19 in Wuhan, China: a retrospective cohort study. Lancet.

[44] Cummings MJ, Baldwin MR, Abrams D (2020). Epidemiology, clinical course, and outcomes of critically ill adults with Covid-19 in New York City: a prospective cohort study. Lancet.

[45] Chen X, Zhao B, Qu Y (2020). Detectable serum Sars-Cov-2 viral load (Rnaaemia) is closely correlated with drastically elevated interleukin 6 (IL-6) level in critically ill Covid-19 patients. Clin Infect Dis.

[46] Yang Y, Shen C, Li J (2020). Plasma Ip-10 and Mcp-3 levels are highly associated with disease severity and predict the progression of Covid-19. J Allergy Clin Immunol.

[47] Chang CC, Ho SC, Wang LY, Yang CY (2007). Bladder cancer in Taiwan: relationship to trihalomethane concentrations present in drinking-water supplies. J Toxicol Environ Health A.

[48] Moutchia J, Pokharel P, Kerri A (2020). Clinical laboratory parameters associated with severe or critical novel coronavirus disease 2019 (Covid-19): a systematic review and meta-analysis. PLoS ONE.

[49] Van Den Berghe G (2014). Non-thyroidal illness in the ICU: a syndrome with different faces. Thyroid.

[50] Maia AL, Goemann IM, Meyer EI, Wajner SM (2011). Deiodinases: the balance of thyroid hormone: type 1 iodothyronine deiodinase in human physiology and disease. J Endocrinol.

[51] Maia AL, Kim BW, Huang SA, Harney JW, Larsen PR (2005). Type 2 iodothyronine deiodinase is the major source of plasma T3 in euthyroid humans. J Clin Investig.

[52] Williams GR, Bassett JH (2011). Deiodinases: the balance of thyroid hormone: local control of thyroid hormone action: role of type 2 deiodinase. J Endocrinol.

[53] Mckeever L, Peterson SJ, Lateef O (2020). Higher caloric exposure in critically ill patients transiently accelerates thyroid hormone activation. J Clin Endocrinol Metab.

[54] Maiden MJ, Torpy DJ (2019). Thyroid hormones in critical illness. Crit Care Clin.

[55] Liu J, Wu X, Lu F, Zhao L, Shi L, Xu F (2016). Low T3 syndrome is a strong predictor of poor outcomes in patients with community-acquired pneumonia. Sci Rep.

[56] Fliers E, Bianco AC, Langouche L, Boelen A (2015). Thyroid function in critically ill patients. Lancet Diabetes Endocrinol.

[57] Pang XP, Harshman JM, Chung M, Pekary AE (1989). Characterization of tumor necrosis factor-alpha receptors in human and rat thyroid cells and regulation of the receptors by thyrotropin. Endocrinology.

[58] Kawabe Y, Eguchi K, Shimomura C (1989). Interleukin-1 production and action in thyroid tissue. J Clin Endocrinol Metab.

[59] Nolte A, Bechtner G, Rafferzeder M, Gärtner R (1994). Interleukin-1 beta (Il-1 Beta) binds to intact porcine thyroid follicles, decreases iodide uptake but has no effect on camp formation or proliferation. Horm Metab Res.

[60] Sato K, Satoh T, Shizume K (1990). Inhibition of 125i organification and thyroid hormone release by interleukin-1, tumor necrosis factor-alpha, and interferon-gamma in human thyrocytes in suspension culture. J Clin Endocrinol Metab.

[61] Yamashita S, Kimura H, Ashizawa K (1989). Interleukin-1 inhibits thyrotrophin-induced human thyroglobulin gene expression. J Endocrinol.

[62] Ak R, Diamant M, Blichert-Toft M, Bendtzen K, Feldt-Rasmussen U (1997). The effects of interleukin-1beta (Il-1beta) on human thyrocyte functions are counteracted by the Il-1 receptor antagonist. Endocrinology.

[63] Ashizawa K, Yamashita S, Tobinaga T (1989). Inhibition of human thyroid peroxidase gene expression by interleukin 1. Acta Endocrinol (Copenh).

[64] Kwakkel J, Wm W, Boelen A (2006). Differential involvement of nuclear factor-kappab and activator protein-1 pathways in the interleukin-1beta-mediated decrease of deiodinase type 1 and thyroid hormone receptor beta1 MRNA. J Endocrinol.

[65] Yu J, Koenig RJ (2006). Induction of type 1 iodothyronine deiodinase to prevent the nonthyroidal illness syndrome in mice. Endocrinology.

[66] Fujii T, Sato K, Ozawa M (1989). Effect of interleukin-1 (Il-1) on thyroid hormone metabolism in mice: stimulation by Il-1 of iodothyronine 5'-deiodinating activity (type I) in the liver. Endocrinology.

[67] DUbuis JM, Dayer JM, Siegrist-Kaiser CA, Gurger AG (1988). Human recombinant interleukin-1 beta decreases plasma thyroid hormone and thyroid stimulating hormone levels in rats. Endocrinology.

[68] Hermus RM, Sweep CG, Van Der Meer MJ (1992). Continuous infusion of interleukin-1 beta induces a nonthyroidal illness syndrome in the rat. Endocrinology.

[69] Wassen FW, Moerings EP, Van Toor H, De Vrey EA, Hennemann G, Everts ME (1996). Effects of interleukin-1 beta on thyrotropin secretion and thyroid hormone uptake in cultured rat anterior pituitary cells. Endocrinology.

[70] Poth M, Tseng YC, Wartofsky L (1991). Inhibition of tsh activation of human cultured thyroid cells by tumor necrosis factor: an explanation for decreased thyroid function in systemic illness?. Thyroid.

[71] Nagaya T, Fujieda M, Otsuka G, Yang JP, Okamoto T, Seo H (2000). A potential role of activated Nf-kappa B in the pathogenesis of euthyroid sick syndrome. J Clin Investig.

[72] Ozawa M, Sato K, Han DC, Kawakami M, Tsushima T, Shizume K (1988). Effects of tumor necrosis factor-alpha/cachectin on thyroid hormone metabolism in mice. Endocrinology.

[73] Perlmutter DH, Dinarello CA, Punsal PI, Colten HR (1986). Cachectin/tumor necrosis factor regulates hepatic acute-phase gene expression. J Clin Investig.

[74] Baur A, Bauer K, Jarry H, Köhrle J (2000). Effects of proinflammatory cytokines on anterior pituitary 5'-deiodinase type I and type II. J Endocrinol.

[75] Feelders RA, Swaak AJ, Romijn JA (1999). Characteristics of recovery from the euthyroid sick syndrome induced by tumor necrosis factor alpha in cancer patients. Metabolism.

[76] Spitzweg C, Joba W, Morris JC, Heufelder AE (1999). Regulation of sodium iodide symporter gene expression in Frtl-5 rat thyroid cells. Thyroid.

[77] Tang KT, Braverman LE, Devito WJ (1995). Tumor necrosis factor-alpha and interferon-gamma modulate gene expression of type I 5'-deiodinase, thyroid peroxidase, and thyroglobulin in Frtl-5 rat thyroid cells. Endocrinology.

[78] Krogh Rasmussen A, Kayser L, Bech K, Feldt-Rasmussen U, Perrild H, Bendtzen K (1991). Influence of interleukin 6 on the function of secondary cultures of human thyrocytes. Acta Endocrinol (Copenh).

[79] Yamazaki K, Yamada E, Kanaji Y (1996). Interleukin-6 (Il-6) inhibits thyroid function in the presence of soluble Il-6 receptor in cultured human thyroid follicles. Endocrinology.

[80] Tominaga T, Yamashita S, Nagayama Y (1991). Interleukin 6 inhibits human thyroid peroxidase gene expression. Acta Endocrinol (Copenh).

[81] Wajner SM, Goemann IM, Bueno AL, Larsen PR, Maia AL (2011). Il-6 promotes nonthyroidal illness syndrome by blocking thyroxine activation while promoting thyroid hormone inactivation in human cells. J Clin Investig.

[82] Bartalena L, Hammond GL, Flink II, Robbins J (1993). Interleukin-6 inhibits corticosteroid-binding globulin synthesis by human hepatoblastoma-derived (Hep G2) cells. Endocrinology.

[83] Ramadori G, Van Damme J, Rieder H, Meyer Zum Büschenfelde KH (1988) Interleukin 6, the third mediator of acute-phase reaction, modulates hepatic protein synthesis in human and mouse. Comparison with interleukin 1 beta and tumor necrosis factor-alpha. Eur J Immunol 18(8):1259–1264. 10.1002/Eji.1830180817 10.1002/eji.1830180817 3138137

[84] Boelen A, Maas MA, Lowik CW, Platvoet MC, Wiersinga WM (1996). Induced illness in interleukin-6 (Il-6) knock-out mice: a causal role of il-6 in the development of the low 3,5,3′-triiodothyronine syndrome. Endocrinology.

[85] Bartalena L, Bogazzi F, Brogioni S, Grasso L, Martino E (1998). Role of cytokines in the pathogenesis of the euthyroid sick syndrome. Eur J Endocrinol.

[86] Davies PH, Black EG, Sheppard MC, Franklyn JA (1996). Relation between serum interleukin-6 and thyroid hormone concentrations in 270 hospital in-patients with non-thyroidal illness. Clin Endocrinol (Oxf).

[87] Friberg L, Werner S, Eggertsen G, Ahnve S (2002). Rapid down-regulation of thyroid hormones in acute myocardial infarction: is it cardioprotective in patients with angina?. Arch Intern Med.

[88] Torpy DJ, Tsigos C, Lotsikas AJ, Defensor R, Chrousos GP, Papanicolaou DA (1998). Acute and delayed effects of a single-dose injection of interleukin-6 on thyroid function in healthy humans. Metabolism.

[89] Weetman AP (1987). Recombinant gamma-interferon stimulates iodide uptake and cyclic amp production by the Ftrl5 thyroid cell line. FEBS Lett.

[90] Kung AW, Lau KS (1990). Interferon-gamma inhibits thyrotropin-induced thyroglobulin gene transcription in cultured human thyrocytes. J Clin Endocrinol Metab.

[91] Kraiem Z, Sobel E, Sadeh O, Kinarty A, Lahat N (1990). Effects of gamma-interferon on DR antigen expression, growth, 3,5,3′-triiodothyronine secretion, iodide uptake, and cyclic adenosine 3′,5′-monophosphate accumulation in cultured human thyroid cells. J Clin Endocrinol Metab.

[92] Misaki T, Tramontano D, Ingbar SH (1988). Effects of rat gamma- and non-gamma-interferons on the expression of IA antigen, growth, and differentiated functions of Frtl5 cells. Endocrinology.

[93] Nagayama Y, Izumi M, Ashizawa K (1987). Inhibitory effect of interferon-gamma on the response of human thyrocytes to thyrotropin (Tsh) stimulation: relationship between the response to Tsh and the expression of Dr antigen. J Clin Endocrinol Metab.

[94] Asakawa H, Hanafusa T, Kobayashi T, Takai S, Kono N, Tarui S (1992). Interferon-gamma reduces the thyroid peroxidase content of cultured human thyrocytes and inhibits its increase induced by thyrotropin. J Clin Endocrinol Metab.

[95] Zakarija M, Hornicek FJ, Levis S, Mckenzie JM (1988). Effects of gamma-interferon and tumor necrosis factor alpha on thyroid cells: induction of class II antigen and inhibition of growth stimulation. Mol Cell Endocrinol.

[96] Caturegli P, Hejazi M, Suzuki K (2000). Hypothyroidism in transgenic mice expressing Ifn-gamma in the thyroid. Proc Natl Acad Sci USA.

[97] Corssmit EP, Heyligenberg R, Endert E, Sauerwein HP, Romijn JA (1995). Acute effects of interferon-alpha administration on thyroid hormone metabolism in healthy men. J Clin Endocrinol Metab.

[98] De Metz J, Romijn JA, Gouma DJ (2002). Interferon-gamma administration does not affect human thyroid hormone metabolism in the post-surgical euthyroid sick syndrome. J Endocrinol Investig.

[99] Carella C, Mazziotti G, Morisco F (2002). The addition of ribavirin to interferon-alpha therapy in patients with hepatitis C virus-related chronic hepatitis does not modify the thyroid autoantibody pattern but increases the risk of developing hypothyroidism. Eur J Endocrinol.

[100] Rotondi M, Chiovato L, Romagnani S, Serio M, Romagnani P (2007). Role of chemokines in endocrine autoimmune diseases. Endocr Rev.

[101] Chen M, Zhou W, Xu W (2020). Thyroid function analysis in 50 patients with Covid-19: a retrospective study. Thyroid.

[102] Lania A, Sandri MT, Cellini M, Mirani M, Lavezzi E, Mazziotti G (2020). Thyrotoxicosis in patients with Covid-19: the Thyrcov study. Eur J Endocrinol.

[103] Wei L, Sun S, Xu CH (2007). Pathology of the thyroid in severe acute respiratory syndrome. Hum Pathol.

[104] Wei L, Sun S, Zhang J (2010). Endocrine cells of the adenohypophysis in severe acute respiratory syndrome (Sars). Biochem Cell Biol.

[105] Li W, Moore MJ, Vasilieva N (2003). Angiotensin-converting enzyme 2 is a functional receptor for the Sars coronavirus. Nature.

[106] Li MY, Li L, Zhang Y, Wang XS (2020). Expression of the Sars-Cov-2 cell receptor gene Ace2 in a wide variety of human tissues. Infect Dis Poverty.

[107] Rotondi M, Coperchini F, Ricci G (2020). Detection of Sars-Cov-2 receptor Ace-2 Mrna in thyroid cells: a clue for Covid-19-related subacute thyroiditis. J Endocrinol Investig.

[108] Brancatella A, Ricci D, Viola N, Sgrò D, Santini F, Latrofa F (2020). Subacute thyroiditis after Sars-Cov-2 infection. J Clin Endocrinol Metab.

[109] Rm R, Campennì A, Siracusa M, Frazzetto G, Gullo D (2020). Subacute thyroiditis in a patient infected with Sars-Cov-2: an endocrine complication linked to the Covid-19 pandemic. Hormones (Athens).

[110] Asfuroglu Kalkan E, Ates I (2020). A case of subacute thyroiditis associated with Covid-19 infection. J Endocrinol Investig.

[111] Ippolito S, Dentali F, Ml T (2020). Sars-Cov-2: a potential trigger for subacute thyroiditis? Insights from a case report. J Endocrinol Investig.

[112] Brancatella A, Ricci D, Cappellani D (2020). Is subacute thyroiditis an underestimated manifestation of Sars-Cov-2 infection? Insights from a case series. J Clin Endocrinol Metab.

[113] Muller I, Cannavaro D, Dazzi D (2020). Sars-Cov-2-related atypical thyroiditis. Lancet Diabetes Endocrinol.

[114] Khoo B, Tan T, Clarke SA (2020). Thyroid function before, during and after Covid-19. J Clin Endocrinol Metab.

[115] Lui DTW, Lee CH, Chow WS (2020). Thyroid dysfunction in relation to immune profile, disease status and outcome in 191 patients with Covid-19. J Clin Endocrinol Metab.

[116] Gao W, Guo W, Guo Y (2020). Thyroid hormone concentrations in severely or critically ill patients with Covid-19. J Endocrinol Investig.

[117] Mateu-Salat M, Urgell E, Chico A (2020). Sars-Cov-2 as a trigger for autoimmune disease: report of two cases of Graves' disease after Covid-19. J Endocrinol Investig.

[118] Tee LY, Harjanto S, Rosario BH (2020). Covid-19 complicated by hashimoto's thyroiditis. Singap Med J.

[119] Shang L, Zhao J, Hu Y, Du R, Cao B (2020). On the use of corticosteroids for 2019-Ncov pneumonia. Lancet.

[120] Russell CD, Millar JE, Baillie JK (2020). Clinical evidence does not support corticosteroid treatment for 2019-Ncov lung injury. Lancet.

[121] Dagens A, Sigfrid L, Cai E (2020). Scope, quality, and inclusivity of clinical guidelines produced early in the Covid-19 pandemic: rapid review. BMJ.

[122] Wang D, Hu B, Hu C (2020). Clinical characteristics of 138 hospitalized patients with 2019 novel coronavirus-infected pneumonia in Wuhan, China. JAMA.

[123] Xu XW, Wu XX, Jiang XG (2020). Clinical findings in a group of patients infected with the 2019 novel coronavirus (Sars-Cov-2) outside of Wuhan, China: retrospective case series. BMJ.

[124] Horby P, Lim WS, Emberson JR (2020). Dexamethasone in hospitalized patients with Covid-19—preliminary report. N Engl J Med.

[125] Wilber JF, Utiger RD (1969). The effect of glucocorticoids on thyrotropin secretion. J Clin Investig.

[126] Brabant A, Brabant G, Schuermeyer T (1989). The role of glucocorticoids in the regulation of thyrotropin. Acta Endocrinol (Copenh).

[127] John CD, christian HC, Morris JF, Flower RJ, Solito E, Buckingham JC (2003). Kinase-dependent regulation of the secretion of thyrotrophin and luteinizing hormone by glucocorticoids and annexin 1 peptides. J Neuroendocrinol.

[128] Cintra A, Fuxe K, Wikström AC, Visser T, Gustafsson JA (1990). Evidence for thyrotropin-releasing hormone and glucocorticoid receptor-immunoreactive neurons in various preoptic and hypothalamic nuclei of the male rat. Brain Res.

[129] Alkemade A, Unmehopa UA, Wiersinga WM, Swaab DF, Fliers E (2005). Glucocorticoids decrease thyrotropin-releasing hormone messenger ribonucleic acid expression in the paraventricular nucleus of the human hypothalamus. J Clin Endocrinol Metab.

[130] Lopresti JS, Eigen A, Kaptein E, Anderson KP, Spencer CA, Nicoloff JT (1989). Alterations in 3,3′5′-triiodothyronine metabolism in response to propylthiouracil, dexamethasone, and thyroxine administration in man. J Clin Investig.

[131] Bianco AC, Salvatore D, Gereben B, Berry MJ, Larsen PR (2002). Biochemistry, cellular and molecular biology, and physiological roles of the iodothyronine selenodeiodinases. Endocr Rev.

[132] Degroot LJ, Hoye K (1976). Dexamethasone suppression of serum T3 and T4. J Clin Endocrinol Metab.

[133] Burch HB (2019). Drug effects on the thyroid. N Engl J Med.

[134] Buijsers B, Yanginlar C, Ml M-H, De Mast Q, Van Der Vlag J (2020). Beneficial non-anticoagulant mechanisms underlying heparin treatment of Covid-19 patients. Ebiomedicine.

[135] Mendel CM, Frost PH, Kunitake ST, Cavalieri RR (1987). Mechanism of the heparin-induced increase in the concentration of free thyroxine in plasma. J Clin Endocrinol Metab.

[136] Stevenson HP, Archbold GP, Johnston P, Young IS, Sheridan B (1998). Misleading serum free thyroxine results during low molecular weight heparin treatment. Clin Chem.

[137] Stockigt JR, Lim CF (2009). Medications that distort in vitro tests of thyroid function, with particular reference to estimates of serum free thyroxine. Best Pract Res Clin Endocrinol Metab.

[138] Sapin R, Jl S, Gasser F (2000). Intermethod discordant free thyroxine measurements in bone marrow-transplanted patients. Clin Chem.

